# Efficacy and safety of duloxetine for postoperative pain after total knee arthroplasty in centrally sensitized patients: study protocol for a randomized controlled trial

**DOI:** 10.1186/s12891-021-04168-x

**Published:** 2021-03-30

**Authors:** Shicheng Wang, Wensheng Wang, Long Shao, Jing Ling

**Affiliations:** 1 Department of Orthopaedic Surgery, Ningbo No.6 Hospital, 1059 Zhongshan East Road, Ningbo, 315040, Zhejiang China; 2 Department of Neurology, Ningbo No.6 Hospital, 1059 Zhongshan East Road, Ningbo 315040, Zhejiang, China

**Keywords:** Central sensitization, Duloxetine, Pain, Total knee arthroplasty

## Abstract

**Background:**

Postoperative residual knee pain after total knee arthroplasty (TKA) is a significant factor that contributes to patient dissatisfaction. Patients with preoperative central sensitization (CS) may be more susceptible to unexplained chronic pain after TKA, and duloxetine has been reported to be effective in post-TKA pain control in patients with CS. However, there remains limited evidence to support this off-label use in routine clinical practice. Hence, we designed this randomized, placebo-controlled, triple-blind clinical trial to evaluate the effects of preoperative screening and targeted duloxetine treatment of CS on postoperative residual pain compared with the care-as-usual control group.

**Methods:**

This randomized controlled trial includes patients with knee osteoarthritis on a waiting list for primary unilateral TKA. Patients with preoperative CS will be randomly allocated to the perioperative duloxetine treatment group (duloxetine group) or the care-as-usual control group (placebo group). Patients in the duloxetine group will receive a half-dose of preemptive duloxetine (30 mg/day) for a week before surgery and a full-dose of duloxetine (60 mg/day) for six weeks after surgery. The primary outcome is the intensity of residual pain at six months after TKA, including the visual analogue scale, 11-point numeric rating scale, the sensory dimension of the brief pain inventory, and the pain subscale of the Knee injury and Osteoarthritis Outcome Score. The secondary outcome measures will include the pain and function related outcomes. All of the patients will be followed up at one, three, and six months after surgery. All adverse events will be recorded and immediately reported to the primary investigator and ethics committee to decide if the patient needs to drop out from the trial.

**Discussion:**

This clinical trial will convey the latest evidence of the efficacy and safety of the application of duloxetine in postoperative pain control in CS patients who are scheduled for TKA. The study results will be disseminated at national and international conferences and published in peer-reviewed journals.

**Trial registration:**

Chinese Clinical Trial Registry (http://www.chictr.org.cn) registration number: ChiCTR2000031674. Registered 07 April 2020.

## Background

Total knee arthroplasty (TKA) is one of the most successful and effective surgical options performed in orthopedics, aiming to relieve pain and restore function for patients with end-stage knee osteoarthritis (OA) [1]. Over the past decades, significant advances have been made in TKA surgical techniques, implant design, patient selection, and perioperative care management, which led to the continuous improvement of clinical outcomes and expanded surgical indications [2–16]. Given the rapidly aging population and the growing prevalence of knee-related diseases such as OA and rheumatoid arthritis, the number of TKA procedures is projected to increase substantially worldwide [17–19]. However, the dissatisfaction rate following a TKA has persisted over the last decade, with approximately 10 %~20 % of patients remain dissatisfied following primary TKA [20–23]. As patient satisfaction has been considered a key parameter in assessing success after TKA, a substantial proportion of patients failed to achieve the desired goal of surgery [22]. Therefore, patient dissatisfaction following TKA remains a challenging problem and should be a high priority.

Previous studies have investigated the causes of dissatisfaction after TKA, and the most common reasons were residual pain and limited function [22, 24–27]. A significant number of patients remain dissatisfied as the persistent chronic pain was not relieved, even though the TKA procedure has removed the pain source [28, 29]. Recent studies found that the pain in patients with OA varies from nociceptive to neuropathic pain-(NP) like symptoms, which may contribute to joint nociceptors’ exposure to the changing biochemical environment during the process of OA [30]. It is thought that these changes might contribute to the activation and sensitization of nociceptors, lead to hyperexcitability of the central nervous system (central sensitization, CS) [29–32]. CS is defined as increased responsiveness of nociceptive neurons in the central nervous system, which is characterized by pain in the presence of a non-noxious stimulus (allodynia) and pain hypersensitivity (hyperalgesia) [33]. Not all patients with knee OA are suffered from CS, but the subgroup of patients with CS is related to low pain thresholds, high level of preoperative pain, and severe pain in the early postoperative period after TKA [34–36]. Furthermore, preoperative CS has been recently recognized as a significant risk factor for persistent pain and dissatisfaction following TKA, which could persist two years after TKA, resulting in worse quality of life, functional disability, and dissatisfaction [28, 34, 37–40]. Considering postoperative residual pain is the primary cause of dissatisfaction following TKA and CS has been identified as a risk factor for persistent postoperative pain and dissatisfaction, preoperative screening and treatment of CS could be an effective way to decrease the level of residual pain after TKA and improve patient satisfaction.

The treatment options for CS focus on those strategies that specifically target the pathophysiological mechanisms known to be involved in CS, which aim to desensitize the central nervous system. The treatment options mainly include pharmacological options (acetaminophen, serotonin-reuptake inhibitor drugs, selective and balanced serotonin and norepinephrine-reuptake inhibitor (SNRI) drugs, serotonin precursor tryptophan, opioids, N-methyl-d-aspartate (NMDA)-receptor antagonists, calcium-channel alpha(2)delta (a2δ) ligands), rehabilitation (e.g., transcutaneous electric nerve stimulation (TENS), manual therapy) and neurotechnology options (e.g., transcranial magnetic stimulation) [41, 42]. Ho et al. performed a randomized controlled trial (RCT) to evaluate the efficacy of duloxetine in reducing morphine requirements in patients after TKA and found that perioperative administration of duloxetine could reduce postoperative morphine requirements during the first 48 h after TKA, without significant differences in pain scores or adverse effects [43]. YaDeau et al. conducted a triple-blinded RCT to evaluate the efficacy of duloxetine on subacute pain after TKA, in which patients received 60 mg/day from the day of surgery for 14 days [44]. They found that duloxetine did not reduce pain at rest, with ambulation or flexion, but duloxetine could reduce opioid consumption and nausea in the first 14 days after surgery [44]. Compared with the normal controls, patients with CS are associated with more severe postoperative pain, greater opioid consumption, and a higher risk of persistent pain, so the analgesic efficacy of peri-TKA duloxetine could be more effective in patients with CS. Koh et al. conducted an RCT in patients with preoperative CS to investigate whether duloxetine could reduce postoperative pain and improve recovery quality after TKA [32]. This study suggested that in patients with preoperatively identified CS, duloxetine reduced postoperative pain and improved quality of recovery, without increasing the risk of adverse medication effects following TKA [32].

Based on previous research, for patients who are identified as having CS before TKA, supplementing perioperative duloxetine to the multimodal analgesic protocol may help to reduce postoperative pain and opioid consumption, improve the quality of recovery in terms of both emotional and physical functioning, without increasing the risk of adverse events [32, 43, 44]. However, at this time, there is insufficient clinical data to recommend routine use of duloxetine in patients with CS to ameliorate the unexplained postoperative pain after TKA. In order to tackle this knowledge gap, we designed this prospective randomized study to evaluate the efficacy and safety of preoperative screening and targeted duloxetine treatment of CS on residual pain compared with the care-as-usual control group. We hypothesized that perioperative duloxetine would reduce postoperative pain and analgesic consumption, and enhance postoperative functional recovery after TKA, without increasing the risk of adverse events in patients who are identified as having CS before TKA. We present the following article in accordance with the *Standard Protocol Items: Recommendations for Interventional Trials (SPIRIT)* reporting checklist [45].

## Methods

### Study design

**Fig. 1:**
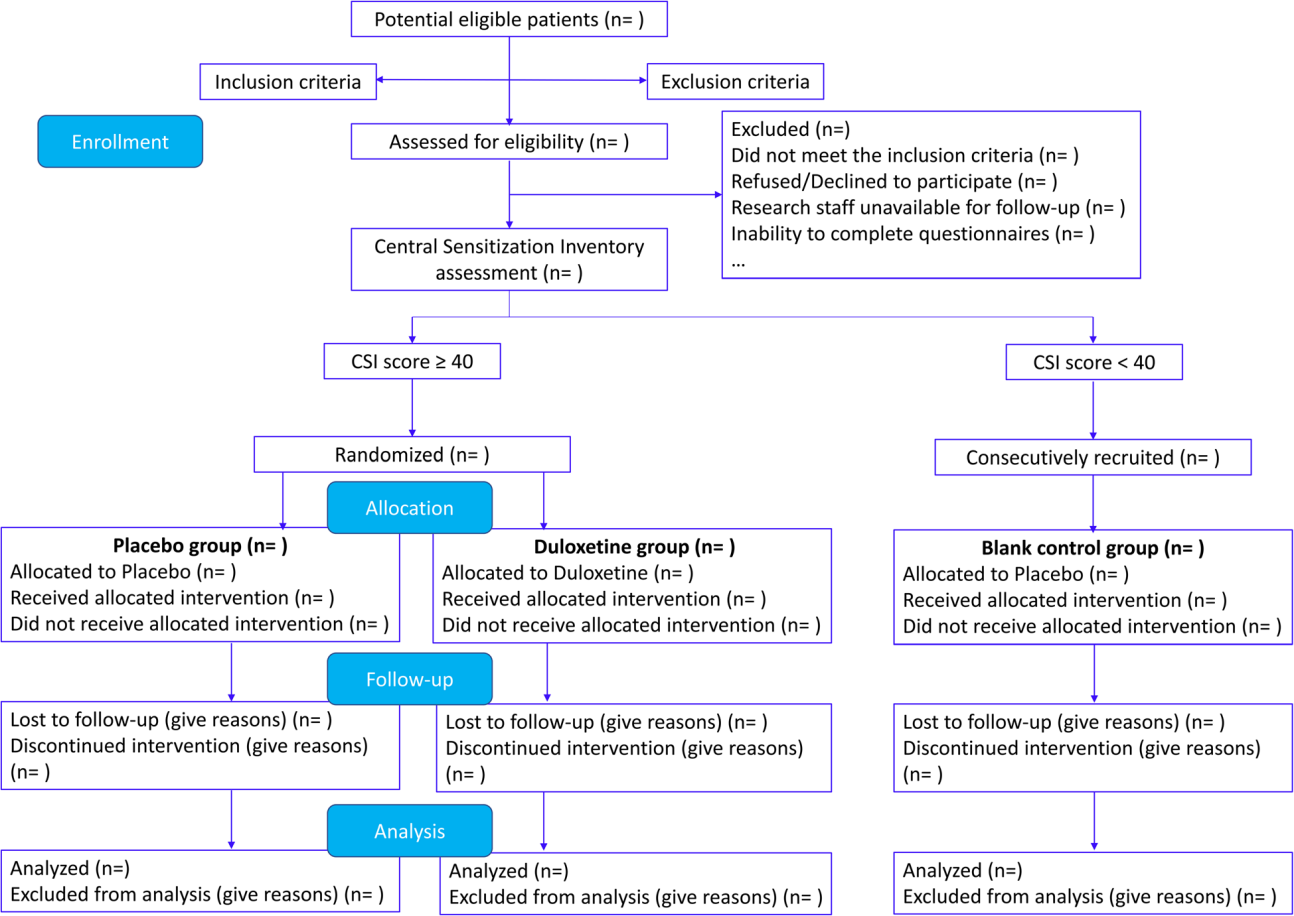
CONSORT 2010 flow diagram of the study sample

This prospective, randomized clinical trial was designed as a single-center parallel-group study with balanced randomization and enrolled patients who are scheduled for TKA. This protocol is reported following the *SPIRIT* statement [45]. This study was approved by our institutional review board and was registered at the Chinese Clinical Trial Registry (http://www.chictr.org.cn) (ChiCTR2000031674). Ethical approval for this retrospective cohort study was obtained from the institutional ethical committee (K2020005). The trial flow chart is shown in Fig. 1.

### Participants

All potentially eligible participants will be selected from the consecutively recruited patients who agreed to participate. According to the inclusion and exclusion criteria, one of the authors will screen the potentially qualified participants.

### Inclusion criteria

Participants who fulfill the following criteria will be included: (I) Patients scheduled to undergo primary unilateral TKA for primary OA of the knee; (II) aged 50 years or older (CS are more prevalent in patients with a long history of knee OA); (III) had education above primary school; (VI) American Society of Anesthesiologists (ASA) physical status grade I ~ III; (V) participants who voluntarily participate in the study and sign a written informed consent form.

### Exclusion criteria

Candidates who meet any of the following criteria will be excluded from participation:

#### General exclusion criteria

(I) Received surgical knee joint procedures in the past year; (II) intra-articular knee injection or knee arthroscopy in the past three months; (III) received other surgical procedure (e.g., hip joint procedure, major thoracic or abdominal operations that may influence the assessment of CS) during the past year; (VI) planned or intended contralateral TKA procedure or any other surgical procedure within the study duration; (V) cognitive or neurological disorders that could strongly affect the questionnaire surveys (e.g., Alzheimer’s disease, dementia); (VI) inability to complete research questionnaires; (VII) with severe preoperative comorbidities that are more likely to be hospitalized during the course of the study or the illness compromises study participation significantly; (VIII) pre-existing pain (except the knee joint pain) and received relevant analgesic treatment; (IX) patients who refused to participate in the study due to stress events; (X) rejection of randomization.

#### Duloxetine‐related exclusion criteria

(I) Previous exposure to duloxetine, non-selective monoamine oxidase inhibitors, tricyclic antidepressants, selective serotonin reuptake inhibitors or SNRIs; (II) allergy to the duloxetine; (III) simultaneously usage of strong cytochrome P450 1A2 (CYP1A2) inhibitors; (IV) impaired liver function, liver cirrhosis, or liver transplantation; (V) severe renal impairment (estimated creatinine clearance less than 30 ml/min), requiring renal dialysis, or renal transplantation; (VI) history of cardiac arrhythmias, cardiac failure, myocardial infarction; (VII) personality disorder, psychiatric disorders or any major depressive disorder (based on Hospital Anxiety and Depression Scale (HADS), the cut-off point score was ≥ 11) [46]; (VIII) chronic gabapentin or pregabalin use (regular use for longer than 3 months), and chronic opioid use (regular use for longer than 3 months); (IX) a history of alcohol or other substance abuse or dependence prior to enrolment; (X) any other possible conditions that are considered inappropriate to participant in this clinical trial.

### Randomization and allocation

After applying the selection criteria, the potentially eligible patients will be screened with the Central Sensitization Inventory (CSI) preoperatively, with a CSI score ≥ 40 indicating CS and a CSI score of < 40 indicating normal [46–48]. Patients with preoperative CS will be randomly allocated (1:1) to the duloxetine group or the placebo group by a computer-generated randomization list. After randomization, there will be a baseline assessment, such as patient demographics and baseline values for outcome measures. In addition, a group of patients who are assessed as normal will receive the placebo (as the blank control group). The hospital pharmacy will prepare indistinguishable capsules containing either duloxetine or placebo for the study. Randomization and allocation will be revealed only after the required number of subjects has been recruited and the data analysis has been completed.

### Blinding

This trial was designed as a pragmatic, randomized, controlled, triple-blinded trial with three parallel arms. All patients, surgeons, clinical investigators who are responsible for data collection, and statisticians will be kept unaware of group assignments until the final data analyses were completed. Although patients will be kept blind to their prescription, the computer-generated randomization table will be provided to the hospital pharmacy. Therefore, our hospital pharmacy will not be blinded to the randomization and will prepare all medications with a single packet of medicine as scheduled. Considering the success of blinding is a fundamental issue in clinical trials, blinding will be further verified with the Bang’s Blinding Index [49]. After the intervention, patients will answer a separate question (“Guess Duloxetine”, “Guess Placebo”, “Don’t Know”) in the blind assessor questionnaire, which attempts to measure the effectiveness of blinding.

### Perioperative care

All TKAs will be performed by the same orthopedic surgeon using the same approach. The routine perioperative analgesic strategy, including preemptive analgesia (celecoxib), intraoperative periarticular cocktail injection, postoperative patient-controlled analgesia (PCA) and celecoxib, and discharge medication (celecoxib). In case of patients suffering unbearable postoperative pain, morphine or other opioids will also be given, the opioid consumption would also be recorded and compared. In the duloxetine group, patients will receive a half-dose of preemptive duloxetine (30 mg/day) for a week before surgery, and a full-dose of duloxetine (60 mg/day) for six weeks after surgery. The maintenance dose (60 mg/day) of duloxetine was chosen based on previous publications [32, 43, 44, 50]. While patients in the placebo group and blank control group will receive placebo for the same period. All enrolled patients will receive the same standard preoperative publicizing and education, intraoperative type of anesthesia, postoperative care, and rehabilitation.

### Follow‐up period

During hospitalization, the basic metabolic panel will be obtained once or more to monitor serum sodium level, as hyponatremia is a duloxetine-induced complication that may occur early on after duloxetine initiation. In our routine clinical practice, patients are advised to return to the outpatient clinic for follow-up evaluation at one, three, and six months after surgery and subsequently every one year after surgery. Thus, in this clinical trial, all of the patients will be followed up at one, three, and six months after surgery. If patients are unwilling or unable to return to the study center for evaluation, they will be interviewed via telephone. Both the hospital and outpatient follow-up evaluation will be conducted by an investigator who is blinded to the experimental groups.

### Criteria for withdrawal

Participants will be informed that they are under no obligation to participate and have the right to withdraw consent for participation in this clinical trial at any point without prejudice, and their routine clinical care and follow-up will not be affected by declining to participate or withdrawing from the trial. Meanwhile, the investigator or regulatory authority can discontinue a patient’s participation in the clinical trial at any time if medically or otherwise necessary. Of note, it is not recommended to discontinue duloxetine abruptly, especially when taking the maintenance dose of 60 mg/day. Therefore, the participant who wishes to discontinue or withdraw must contact the investigator to obtain discontinuation advice.

### Outcome measurement

The following outcome parameters and patient characteristics will be retrieved from electronic hospital information systems or medical records, physical examination, and patient questionnaires.

### Primary outcome measures

The primary outcome is the intensity of residual pain at six months after TKA. The perception of residual pain will be measured with the visual analogue scale (VAS), 11-point numeric rating scale (NRS), the sensory dimension of the Brief Pain Inventory (BPI), and the pain subscale of the Knee injury and Osteoarthritis Outcome Score (KOOS) at six months after surgery [51–54]. These questionnaires have been proven to be valid and reliable, and the results of these questionnaires will help to ascertain the robustness of our results. The key postoperative time point of six months was chosen as this is recognized as the first possible time point to evaluate the ‘success’ of TKA in clinical practice.

### Secondary outcome measures

The secondary outcome measures will include the following. The pain-related outcomes mainly included the cumulative PCA consumption during the first 48 h after TKA and the amounts of rescue analgesic at 48 h after surgery. We will also evaluate the pain severity using the VAS score, NRS score, BPI score, and KOOS score. The function-related outcomes included knee range of motion (ROM), Knee Society Score (KSS) score, Western Ontario and McMaster Universities Arthritis Index (WOMAC) score, and physical activity [55]. These outcomes will be assessed by means of several questionnaires at multiple follow-up time points. Furthermore, we will also collect other parameters, including joint-associated problems, health-related quality of life, CSI score, depressive and anxiety symptoms, perceived improvement and arthroplasty-related expectations, and patient satisfaction (ordinal scale) [56].

### Patient characteristics

The following baseline descriptive data will be obtained: age, gender, height (cm) and weight (kg), body mass index (BMI), marital status, family status, education level, employment status, duration of OA pain symptoms, Kellgren-Lawrence (K-L) grade, OA pain-related medication consumption, ASA classification, smoking and alcohol consumption, disease history, comorbidities, history of drug use, and past health problems.

### TKA-related characteristics

The surgical procedure-related data include the type of anesthesia, surgical approach, type of implant, operation time, intraoperative and postoperative blood loss, and arthroplasty-related complications.

### Adverse events

All adverse events reported spontaneously by the patients or observed by the investigators or staff will be recorded. If any adverse event occurs, the doctor will provide the corresponding treatment to the patient. Meantime, the adverse events will be immediately reported to the primary investigator and ethics committee to decide if the patient needs to withdraw from the trial. Based on previous literature, the most common adverse events were decreased appetite, nausea and vomiting, fatigue, insomnia, constipation, and dry mouth, which could be well managed with i.v. ondansetron [32, 43, 44].

### Data collection and management

The demographic and baseline characteristic data will be collected by screeners when the patients are recruited and enrolled in this trial (Table 1). During the hospitalization and follow-up periods, the clinical outcomes, questionnaires, incidence of complications, and adverse events will be collected by an independent trained investigator and will be monitored by an independent Data Monitoring Committee. No additional participants will be included during the re-evaluation period. Personal data will be handled confidentially. Every participant will receive a unique code, and data of each patient will be collected under this unique code. A unique patient identification list will be used to link the data to the patient, and the key to the code will be safeguarded by the principal investigator. All source documents will be entered into the trial database (OpenClinica clinical trials software).

**Table 1:** Demographic and preoperative clinical characteristics of the patients

Demographics	Placebo group	Duloxetine group	Blank control group
Age (yr)			
Gender, n (%)			
Male			
Female			
BMI (kg/m^2^)			
Marital status			
Family status			
Education level, n (%)			
Primary school			
High school			
Some college			
Technical degree/associate’s degree			
Bachelor’s degree			
Advanced/professional degree (MA, PhD, etc.)			
Employment status			
ASA status			
I			
II			
III			
Preoperative parameters			
Duration of OA pain symptoms			
Kellgren-Lawrence grade			
OA pain-related medication consumption			
Smoking consumption			
Alcohol consumption			
History of drug use			
CSI score			
VAS score			
NRS score			
BPI-pain severity score			
KOOS score			

*ASA* American Society of Anesthesiologists, *BMI* Body Mass Index, *BPI* Brief Pain Inventory, *CSI* Central Sensitization Inventory, *KOOS* Knee injury and Osteoarthritis Outcome Score, *MA* Master of Arts, *NRS* numeric rating scale, *OA* Osteoarthritis, *VAS* visual analogue scale

### Sample size

Sample size calculation was performed with the VAS score as the primary outcome measure. Based on the previous study, the smallest change score for the VAS score to be considered clinically relevant is 2 points (on a 0–10 scale) between the duloxetine group and the placebo group [32]. The power calculation is performed based on the VAS score difference using a two-sided hypothesis test at an alpha level of 0.05 and a power of 80 %, and a total of 38 participants is needed in each group [57–59]. Taking into account the possibility of 20 % violators or dropouts, we will include 50 patients in each group.

### Statistical analysis

Analyses will be performed using the IBM SPSS software (version 21.0, IBM Corp., New York, NY, USA). All outcomes measures will be assessed using both the ITT (intention-to-treat, all randomly assigned patients) and PP (per-protocol, patients who completed the trial without any protocol deviations) data sets. The missing value will be imputed by the last-observation-carried-forward (LOCF) method. For continuous variables and descriptive values, means ± standard deviations (SDs) will be reported. For the enumerative variables, frequency and corresponding percentage will be calculated. For the variables with a normal distribution, statistical comparisons between the groups will be made by using a *t*-test. For variables with a non-normal distribution or ordinal level, the statistical comparison will be made using the Mann-Whitney U test. Outcomes with a discrete distribution will be expressed as percentages and analyzed by the *chi*-squared test or Fisher’s exact test as appropriate. A *P* value < 0.05 is considered statistically significant.

## Discussion

Residual pain is the most common reason for patient dissatisfaction following TKA. Recently, several studies suggested that CS is associated with chronic postoperative pain and decreased satisfaction after TKA. Duloxetine has been used in an off-label way to treat centrally mediated chronic pain in patients with preoperative CS, but the efficacy of pain control after TKA remains unclear. Currently, the available evidence is extremely limited; thus, we designed this clinical trial to examine the efficacy and safety of duloxetine for postoperative pain after TKA in CS patients. The present study was designed based on previous literature, thus, our trial has some advances and strengthens when compared with previous similar RCTs [32]. First of all, except the duloxetine group and placebo group, we set an extra blank control group, which consists of normal patients without medication. Parents in the blank control group will provide the standard baseline for the trial, which will help to evaluate the magnitude of improvement. Second, several parameters will be included to measure the intensity of residual pain, including the VAS score, NRS score, BPI score, and KOOS score. The consistency of these parameters will help to ascertain the robustness of the results. Third, based on previous RCTs, we will collect all of the efficacy and safety related parameters, which will contribute to a more comprehensive evaluation of duloxetine.

We will ensure that this study is a genuinely randomized, controlled, triple-blinded trial through the full implementation of randomization, blindness, and concealment. We will conduct and report this clinical trial in strict accordance with the Consolidated Standards of Reporting Trials (CONSORT) [60]. It is important to acknowledge that the results of this trial will provide scientific and rigorous clinical evidence for the application of perioperative duloxetine in CS patients being scheduled for TKA.

## Data Availability

Not applicable.

## Abbreviations

ASA: American Society of Anesthesiologists
BMI: Body Mass Index
BPI: Brief Pain Inventory
CONSORT: Consolidated Standards of Reporting Trials
CS: Central Sensitization
CSI: Central Sensitization Inventory
HADS: Hospital Anxiety and Depression Scale
ITT: Intention-To-Treat
K-L: Kellgren-Lawrence
KOOS: Knee injury and Osteoarthritis Outcome Score
KSS: Knee Society Score
LOCF: Last-Observation-Carried-Forward
MA: Master of Arts
NRS: Numeric Rating Scale
NMDA: N-methyl-d-aspartate
OA: Osteoarthritis
PCA: Patient-Controlled Analgesia.
PP: Per-Protocol.
RCT: Randomized Controlled Trial.
ROM: Range Of Motion
SD: Standard Deviation
SNRI: Selective and balanced serotonin and Norepinephrine-Reuptake Inhibitor
SPIRIT: Standard Protocol Items Recommendations for Interventional Trials
TENS: Transcutaneous Electric Nerve Stimulation
TKA: Total Knee Arthroplasty
VAS: Visual Analogue Scale
WOMAC: Western Ontario and McMaster Universities Arthritis Index
